# Regulation of intestinal flora by *Suaeda salsa* extract ameliorates hyperglycemia in a mouse model of type 2 diabetes mellitus

**DOI:** 10.3389/fnut.2024.1499196

**Published:** 2024-12-16

**Authors:** Xuemei Yin, Yinzi Sui, Zhengyan Chu, Suqing Han, Xiaodong Ge, Tingting Liu, Feng Zeng, Ligen Chen, Rong Shao, Wei Xu

**Affiliations:** ^1^College of Marine and Bioengineering, Yancheng Institute of Technology, Yancheng, China; ^2^Clinical Pharmacy Department, Yancheng Second People’s Hospital, Yancheng, China; ^3^College of Food Science, Fujian Agriculture and Forestry University, Fuzhou, China

**Keywords:** *Suaeda salsa* extract, T2DM, hyperglycemia, metabolic dysfunction, intestinal flora

## Abstract

**Introduction:**

Type 2 diabetes mellitus (T2DM) often leads to elevated blood glucose levels and lipid metabolism disorder, which is generally accompanied by dysbiosis of gut microbiota and metabolic dysfunction.

**Methods:**

In this study, a mouse model of T2DM was established by feeding a high-fat/sucrose diet combined with injecting a low dose of streptozotocin. The aim of this study was to analyze the regulatory effect of Suaeda salsa extract (SSE) on T2DM and its effect on the intestinal flora of mice.

**Results:**

The results showed that SSE could significantly improve the body weight, fasting blood glucose (FBG), area under the curve (AUC) of the oral glucose tolerance test (OGTT), glycosylated serum protein (GSP) and islet function index. Moreover, 4-week body weight, FBG, AUC of OGTT, GSP, as well as intestinal acetic and butyric acid were significantly better in the SSE-L than in the MET group (*p* < 0.05). In addition, it was also found that the potential hypoglycemic mechanism of SSE was related to the expression of Akt serine/threonine kinase (AKT-1) and glucose transporter-2 (GLUT-2) genes. Compared with the model group, SSE intervention significantly increased the abundance of probiotics, such as *Soleaferrea*, *Alloprevotella*, *Lactobacillus* and *Faecalibaculum*, while decreasing the relative abundance of harmful bacteria, such as *Phocaeicola* and *Bilophila*. Analysis of the correlation among intestinal microbiota, short chain fatty acids (SCFAs) and the hypoglycemic index showed that *Dwaynesavagella* was significantly correlated with acetic, propionic and butyric acid, as well as all the diabetes-related indexes analyzed in this study.

**Discussion:**

Thus, this taxon can potentially be used as a microbiological marker of type 2 diabetes. Taken together, these findings demonstrate that SSE can alleviate T2DM and its complications by improving glycemia-related indicators and modulating the structure of intestinal flora.

## Graphical abstract

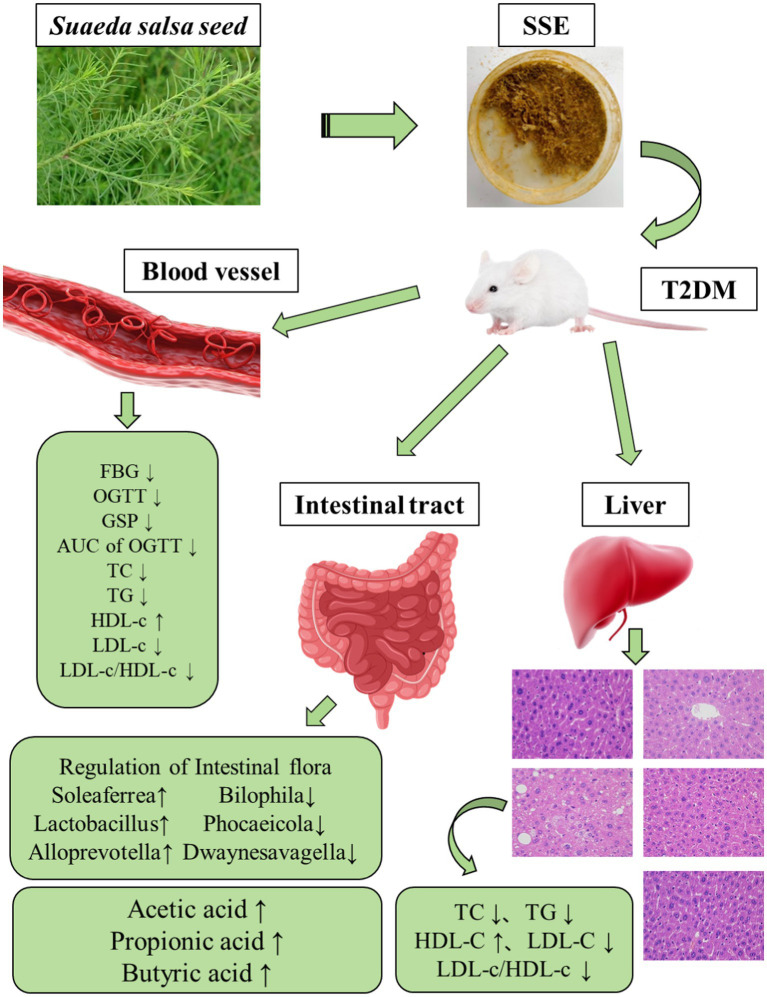

## Highlights

- SSE can improve blood glucose related indexes in T2DM mice.
- SSE regulates islet function index and promotes islet secretion in T2DM mice.
- SSE can regulate the intestinal flora structure associated with T2DM.

## 1 Introduction

Diabetes is a clinical syndrome characterized by chronic hyperglycemia and dyslipidemia. Type 2 diabetes mellitus (T2DM) is a common type of diabetes, and its incidence is increasing around the globe. According to the International Diabetes Federation, 536.6 million adults worldwide were affected by diabetes in 2021, and the total number of adults with diabetes was expected to surpass 780 million by 2045 (1). The World Health Organization predicted that diabetes will become the seventh leading cause of death by 2030 (2). The pathogenesis of type 2 diabetes is complex, encompassing genetics, lifestyle, environmental factors and other unknown influences, resulting in reduced ability of insulin to regulate blood sugar, which is accompanied by functional defects of pancreatic islet beta cells. It should be noted that diabetes is not a single disease, but a spectrum of related diseases. Studies have reported that hyperglycemia is the main cause of diabetic complications (3), as long-term high blood sugar levels will damage the kidneys, cardiovascular system, nerves, bones, eyes and other organs, eventually leading to serious complications (4). As the incidence of the disease continues to increase, the costs associated with diabetes and its complications are becoming a major concern for global public health (5). The traditional treatment of DM is mainly based on synthetic drugs, but all of them are associated with a certain degree of toxic side effects and eventual therapy resistance, which limits their therapeutic effect on DM and increases the occurrence of complications. Plant derived secondary metabolites are characterized by chemical diversity, structural complexity, high biological activity and low toxic side effects. As a consequence, they have recently become the main sources of new drugs, functional foods and food additives. Therefore, finding safe and effective hypoglycemic active ingredients from plants has become an important way to prevent and treat diabetes and its complications (6).

In recent years, active ingredients extracted from plants have been effectively used to prevent or treat metabolic diseases, including T2DM, hyperlipidemia, and hypertension (7). Active ingredients extracted from plants using ethanol have gained increasing attention due to their effectiveness in the regulation and prevention of metabolic disorders (8, 9). Cai (10) found that ethanolic extract of propolis (EEP) could significantly reduce the weight gain of mice fed a high-fat diet (HFD), liver fat accumulation and the formation of proinflammatory cytokines (TNF-α, IL-1β and IL-6), while also reducing insulin resistance as well as normalizing glucose and lipid metabolism. At the same time, EEP modulated the intestinal microbiota and function of HFD-fed mice. The abundance of anti-inflammatory bacteria (*Roseburia* sp., *Intestinimonas* sp., *Parabacteroides goldsteinii*, and *P. distasonis*) in the intestinal flora of mice was increased, while the abundance of proinflammatory bacteria was reduced. Moreover, the dominant flora of EEP treated mice was significantly correlated with obesity, insulin resistance and glycolipid metabolism. Yan et al. (11) found that compared with the model group, fasting blood glucose (FBG), serum fasting insulin (FINS), insulin sensitivity index (HOMA-IS) and insulin resistance index (HOMA-IR) of rats in the extract-treated groups were significantly decreased, while high density lipoprotein (HDL) was significantly increased (*p* < 0.05). Maeda et al. (12) showed that alcoholic extract of garlic significantly improved fatty liver and insulin resistance, while also modulating the gut microbiota in T2DM model mice, with increased relative abundance of *Bifidobacterium*, *Clostridium* cluster XVIII, and *Prevotella*. *Bifidobacterium* is a common genus of beneficial bacteria found in many fermented products and dietary supplements (13). Studies suggest that ethanolic extracts of medicinal plants may have potential benefits to improve T2DM by altering the community and abundance of host gut microbiota, especially by promoting the proliferation of intestinal probiotic bacteria, inhibiting the growth of harmful bacteria, and regulating the abundance of intestinal microbial metabolites. Short chain fatty acids (SCFAs) produced by gut microbiota (GM) play an important role in maintaining intestinal barrier function and blood glucose stability, as well as regulating lipid metabolism and liver glycogen metabolism. As the main energy source of colonic cells, butyric acid plays an important role in maintaining colon homeostasis. Dysfunction of pancreatic islet beta cells is common in T2DM patients, which may be a stress manifestation due to chronic inflammation and impaired metabolic function. Butyric acid was found to significantly increase the expression of the insulin gene and the number of beta cells, but acetic and propionic acid had no such effect. It is believed that butyric acid increased the number of β-cells mainly through its inhibitory effect on class I histone deacetylases (HDACs) (14). Acetic acid was found to inhibit lipid deposition in the liver, skeletal muscle, and adipose tissue (15). propionic acid was found to reduce abdominal fat and the amount of lipids in the liver (16).

*Suaeda salsa* is a well-known salt and alkali-resistant, succulent halophyte in the family *Amaranthaceae*, which was first recorded in an ancient Chinese book “Jiu Huang Ben Cao” that enrolled the potential food plants to cope with famine during Ming dynasty. The nutritional value of *Suaeda salsa* is also very high, as it is rich in various components beneficial to human health, such as fatty acids, flavonoids, crude fiber, and minerals. The active ingredients of *Suaeda salsa* include polyphenols, flavonoids, and polysaccharides, which can scavenge free radicals, reduce cholesterol, and also help patients improve the health of the brain and cardiovascular system (17). Therefore, it has found various applications in food, medicine and other fields. At present, studies on active polysaccharides are limited to their physiological effects. Li et al. (18) isolated and purified polysaccharides from *Suaeda salsa* and obtained the polysaccharide SSP-1, which exhibited antitumor activity and could effectively inhibit the growth of HepG2 tumor cells. Moreover, SSP-1 was not toxic, as it had no significant effect on the viability of human normal liver cell line L-02. Wang (19) found that the superoxide anion scavenging activity of the polysaccharides was higher than that of vitamin C, while the DPPH scavenging activity was comparable. However, the regulatory effect of *Suaeda salsa* extract (SSE) on intestinal microbiota in T2DM mice has not been investigated before. In this study, we established a T2DM mouse model by feeding a high-sugar and high-fat (HSHF) diet, combined with intraperitoneal injection of streptozotocin, and then assessed the effects of SSE on lowering blood glucose and regulating the intestinal microbial community of T2DM mice. The results of this study provided a basis for further exploration of the hypoglycemic effects of SSE.

## 2 Materials and methods

### 2.1 Preparation and characterization of SSE

The extraction method of SSE is based on previous research (20, 21), with minor modifications as follows. Firstly, the seeds of the *Suaeda salsa* plants were naturally dried and passed through a 40-mesh sieve to remove impurities. Then they were suspended in 70% (v/v) ethanol (1:35 w/v), and subjected to heated reflux extraction at 70°C for 5 h. The resulting mixture was filtered, concentrated and freeze-dried to obtain the SSE, which was used in subsequent animal experiments. The main chemical constituents of SSE were identified by UPLC-QTOF-MS/MS (Thermo Fisher Scientific, Waltham, United States) as described before.

### 2.2 Animal experiments

Sixty male ICR mice (SPF, 4 weeks old, 21 ± 2 g) were provided by Jiangsu Medical College (Yancheng, China) (approval number: SYLL-2023-702). The rearing environment was kept at 23 ± 1°C, and the relative humidity was 55 ± 3%. All animals were fed a maintenance diet for 1 week, after which 12 randomly selected mice were placed on a normal diet and the remaining 48 mice were assigned to a high sugar and high fat diet (HSHF; 15% lard, 15% sucrose, 1% cholesterol, 10% yolk, 0.2% sodium deoxycholate, and 58.8% standard chow) for 4 weeks. After fasting for 8–12 h, the HSHF group was injected peritoneally with streptozotocin (STZ, dissolved in 0.1 M citrate buffer, pH 4.5) at dose of 45 mg/kg. The NC group was injected with the same volume of sodium citrate buffer. After 4 days of continuous intraperitoneal injection, the fasting blood glucose (FBG) levels of 60 mice ≥11.1 mmol/L, we considered all the mice were successfully modeled, and all the mice could be regarded as diabetic mice. They were subdivided into four groups according to the principle of similar blood glucose values, including the model group (*n* = 12), metformin group (MET, 100 mg/kg/d, *n* = 12) (22), low-dose SSE group (SSE-L, 100 mg/kg/d, *n* = 12), and high-dose SSE group (SSE-H, 300 mg/kg/d, *n* = 12) (23).

### 2.3 Sample collection and biochemical analysis

The mice in the normal and model groups were given the same amount of deionized water (100 mg/kg/d) once a day according to the indicated dose for 4 consecutive weeks. The fur, autonomous activity and mental state of the mice were observed daily, as well as the daily feed and water intake of the mice were recorded. During intragastric administration, the mice were fasted for 12 h at 0, 2, and 4 weeks, and their body weight was recorded. The FBG level was measured by tail tip blood sampling. In addition, at the end of the 4th week, all the mice were fasted for 12 h and the FBG level (G0 h) was measured. Then, the mice were given glucose solution via intragastric lavage (2 g/kg), the blood glucose levels were measured at 0.5 h, 1 h, and 2 h (G0.5 h, G1 h, and G2 h respectively). Finally, the oral glucose tolerance test (OGTT) was administered and the area under the curve (AUC) was calculated to assess the degree of blood glucose change during OGTT according to the following formula: AUC of OGTT = 0.25 × (G0 h + G0.5 h) + 0.25 × (G0.5 h + G1 h) + 0.5 × (G1 h + G2 h).

All mice were fasted for 12 h before dissection, feces were collected, shock-frozen in liquid nitrogen and stored at −80°C. The blood was taken from the eyeball after anesthesia with ether, and centrifugation at 3,000 rpm for 10 min to harvest the serum. The mice were killed by cervical vertebrae dislocation, liver tissue and cecum contents were dissected, and tissue samples were placed into 4% paraformaldehyde solution at room temperature. The remaining tissues were frozen in liquid nitrogen and stored at −80°C. In addition, the glycated serum proteins (GSP), total cholesterol (TC), triglycerides (TG), high/low-density lipoprotein-cholesterol (HDL-c and LDL-c) were measured using commercial assay kits (Jiancheng, Nanjing, China). The homeostasis model assessment-β index (HOMA-β), HOMA-insulin resistance index (HOMA-IR), and HOMA-insulin sensitivity index (HOMA-IS) (24, 25) were calculated as follows:


$$\begin{array}{l} \mathrm{HOMA}-\mathrm{pancreatic islet}\;\beta \;\mathrm{cell function}\;\left(\mathrm{HOMA}-\beta \right)=\\ 20\times \mathrm{FINS}/\left(FBG-3.5\right) \end{array}$$

$$\mathrm{HOMA}-\mathrm{insulin resistance}\;\left(\mathrm{HOMA}-IR\right)=\mathrm{FINS}\times FBG/22:5$$

$$\mathrm{HOMA}-\mathrm{insulin sensitivity}\;\left(\mathrm{HOMA}-\mathrm{IS}\right)=1/\left(\mathrm{FINS}\times FBG\right)$$

### 2.4 Histopathological analysis

The liver tissues were fixed overnight with 4% paraformaldehyde, embedded in paraffin (Nikon, Japan) and cut into 4 μm thick sections using a microtome (Nikon, Japan). The sections were stained with hematoxylin and eosin (H&E), and the morphology of the liver tissue was observed using a conventional optical microscope (Nikon, Japan) at 400-fold magnification. Pathological scores of liver tissue were calculated according to the criteria described by Kleiner et al. (26).

### 2.5 Quantitative PCR analysis

Total RNA from 30 samples (6 for each group) was reverse/transcribed into cDNA using the PrimeScript^™^ RT Master Mix (Vazyme Biotech Co., Ltd). The qPCR was performed using an Applied Biosystems 7500 Real-Time PCR System (Life Technologies, Gaithersburg, MD, United States). The reactions were carried out in 20 μL volumes containing 1 μL of cDNA, 10 μL of SYBR Premix Ex Taq polymerase (2×) (Vazyme, Nanjing, China), 0.4 μL of ROX Reference Dye II (50×), 0.4 μL of the forward primer (10 mmol/L), 0.4 μL of the reverse primer (10 mmol/L), and 6.8 μL of ddH2O. The temperature program encompassed initial denaturation at 95°C for 30 s, followed by 40 cycles of denaturation 95°C for 5 s and annealing at 60°C for 34 s, at which point fluorescence was measured. Finally, a dissociation curve to test PCR specificity was generated by one cycle at 95°C for 15 s followed by 60°C for 1 min and ramped to 95°C with concomitant fluorescence measurement. Specific primers were designed based on sequences retrieved from the National Center for Biotechnology Information (NCBI) database (Table 1). The β-actin gene was selected as the internal reference for normalization based on the geometric means of the target and reference genes. The $-2^{\Delta \Delta C_{T}}$ method was used to transform the data for relative quantification (27).

**Table 1:** Primers used for quantitative real-time PCR.

Gene name	Forward primer (5′–3′)	Reverse primer (5′–3′)
AKT-1	ACTCATTCCAGACCCACGAC	CCGGTACACCACGTTCTTCT
GLUT-2	TACGGCAATGGCTTTATC	CCTCCTGCAACTTCTCAAT
β-actin	TGTCCACCTTCCAGCAGATGT	AGCTCATAACAGTCCGCCTAGA

### 2.6 Analysis of cecal microbiota

Samples comprising 0.1 g cecal content of each mouse were weighed and the microbial genomic DNA was extracted using the QIAamp-DNA stool mini kit (Qiagen, Hilden, Germany). PCR amplification of the V3–V4 region of the 16S rRNA gene was performed using specific primers 341F (5′-CCTAYGGGRBGCASCAG-3′) and 806R (5′-GGACTACNNGGGTATCTAAT-3′). The PCR reaction conditions encompassed predenaturation at 95°C for 3 min, followed by 30 cycles of denaturation at 98°C for 20 s, annealing at 58°C for 15 s, and extension at 72°C for 20 s, with a final extension at 72°C for 5 min. The PCR products were detected by 2% agarose gel electrophoresis, and the amplicon was purified using AxyPrep DNA gel recovery kit. The purified products were sent to Sangon (Shanghai, China) for sequencing. The sequencing library was prepared, and the PE250 sequencing analysis was performed on an Illumina NovaSeq platform. After splicing and quality control of the original data, operational taxonomic units (OTUs) were generated, and analyzed using the bioincloud technology platform.^1^

### 2.7 Determination of cecal SCFAs

According to the method described in Han et al. (28), the acetic, propionic and butyric acid standard were weighed and filled with ether to a constant volume to form a single standard stock solution, which was diluted with ether into 6 working standard solutions according to the gradient, and the standard curve corresponding to each short-chain fatty acid was fitted according to the corresponding peak areas. For cecal analysis, 100 mg of cecal contents was weighed, placed into a 1.5 mL centrifuge tube, and mixed well with 200 μL of distilled water, followed by ultrasonic extraction for 30 min. The supernatant was then obtained by centrifugation at 3,000 rpm and 4°C for 10 min, diluted with water (1:20), and filtered through a 0.22 μm poresize membrane. Finally, gas chromatography was performed on a instrument (Shimadzu, Kyoto, Japan) equipped with an HP-INNOWax capillary column (30 m × 0.25 mm × 0.25 μm, Agilent, United States). The sample (1 μL) was injected at a shunt ratio of 10:1. The inlet temperature was 250°C and the detector temperature 250°C. The temperature program encompassed an initial temperature of 100°C for 1 min, ramp to 200°C at 5°C/min and hold for 2 min. The carrier gas was helium and a flow rate of 1 mL/min.

### 2.8 Statistical analysis

All experiments were repeated at least three times. The data are shown as means ± standard deviations. Statistical significance was determined using the Kruskal–Wallis test followed by Dunn’s posthoc test with Bonferroni’s correction. A *p*-value <0.05 was considered to indicate statistical significance. All statistical analyses were performed using SPSS 20.0 (IBM, New York, United States).

## 3 Results

### 3.1 Composition of SSE

In this study, 15.34 g SSE could be extracted from 100 g of seeds. A total of 1,059 chemical constituents (ESI^+^: 557, ESI^−^: 502) were detected by UPLC-QTOF/MS, we screened the top 100 compounds in abundance under positive and negative ion conditions, among which the main bioactive components included Benzene and substituted derivatives, such as salicyluric acid, terfenadine, oxymetazoline and aniline; carboxylic acids and derivatives, such as betaine, vinblastine, and domoic acid. In addition, there were a number of Fatty acyls such as 5(S),14(R)-lipoxin b4, cis,cis-muconic acid, N-palmitoyltaurine, nonanoic acid and cis-9-palmitoleic acid. There were also some of flavonoids, such as eriodictyol and apigenin 7,4′-dimethyl ether. The software also deduced a number of phenols, such as bilobol, capsaicin, 3-methylcatechol and DL-octopamine. Steroids and steroid derivatives included pregnenolone, beta-estradiol and estrone sulfate (Supplementary Table S1). The positive and negative ion UPLC-QTOF-MS/MS profile were shown in Supplementary Figures S1, S2.

### 3.2 Effects of SSE on body weight and blood glucose in T2DM model mice

**Figure 1:**
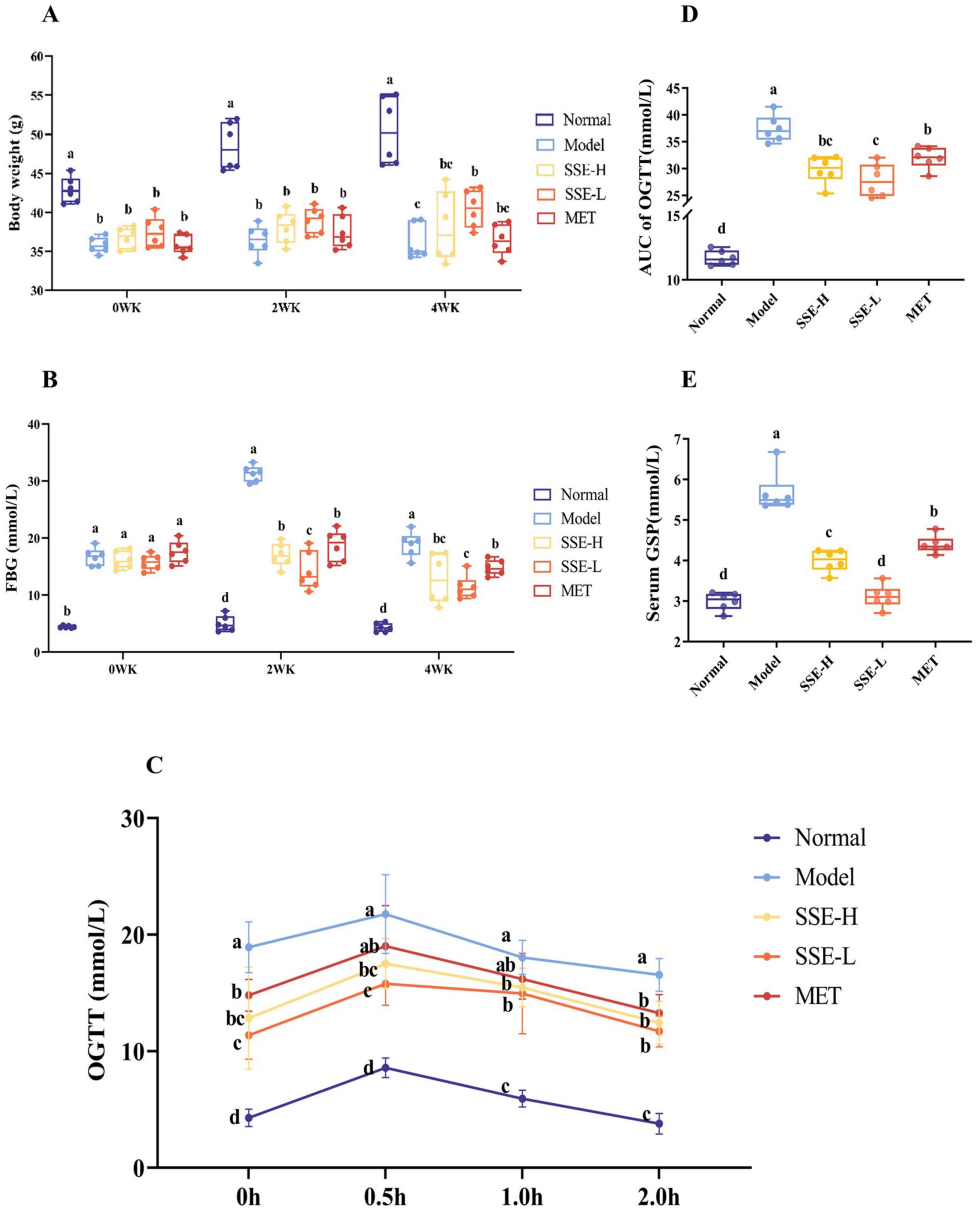
The effects of SSE on **(A)** body weight, **(B)** FBG, **(C)** OGTT, **(D)** AUC of OGTT, and **(E)** GSP in T2DM model mice. (SSE, *Suaeda salsa* extract; FBG, fasting blood glucose; OGTT, oral glucose tolerance test; AUC, area under the curve; GSP, glycosylated serum proteins; T2DM, type 2 diabetes mellitus; WK, week. Different superscript letters indicate statistically significant differences between the groups, *p* < 0.05).

As shown in Figure 1A, in the first week, the body weight of mice in all treatment groups significantly decreased compared to the normal group (*p* < 0.05). When T2DM mice were treated with different doses of SSE for 2 weeks, the body weight was still significantly decreased compared with the normal group (*p* < 0.05). After 4 weeks of SSE treatment, the body weight in was still significantly decreased compared with the normal group (*p* < 0.05), but compared with the model group, the body weight of mice in the SSE-L group significantly increased (*p* < 0.05). Figure 1B shows the change trend of fasting glucose levels in T2DM mice during the whole SSE treatment period. In the first week, FBG level of all treatment groups was significantly increased compared with the normal group (*p* < 0.05). After T2DM mice were treated with SSE for 2 weeks, the FBG of the SSE-L, SSE-H and MET groups was significantly higher than in the normal group (*p* < 0.05), but it was significantly lower than in the model group (*p* < 0.05). In addition, the FBG of the SSE-L group was significantly lower than that of the SSE-H and MET groups (*p* < 0.05). After 4 weeks of SSE treatment, the FBG was still significantly higher in the SSE-L and SSE-H groups than in the normal group (*p* < 0.05). By contrast, the FBG of the SSE-L, SSE-H, and MET groups significantly decreased compared with the model group (*p* < 0.05). Moreover, the FBG of the SSE-L group was significantly lower than that of the MET group (*p* < 0.05). Next, the glucose loading capacity of T2DM mice was investigated, as shown in Figure 1C. When the mice were administered 2 g glucose per kg body weight, their blood glucose levels quickly rose to the highest point and then showed a gradual downward trend. The initial blood sugar level of the normal group was significantly lower than in the other groups (*p* < 0.05), while that of the model group was significantly higher (*p* < 0.05). At 0.5 h, the blood glucose levels of all groups reached the highest point, but those of the SSE-L, SSE-H and MET groups were significantly lower than in the model group (*p* < 0.05). At 1 and 2 h, the blood glucose levels of all groups showed a gradual downward trend, but remained elevated in the SSE-L, SSE-H and MET groups compared to the normal group (*p* < 0.05). Nevertheless, they were significantly lower in all treatment groups than in the untreated model group (*p* < 0.05). In order to more intuitively evaluate the ability of the mice to regulate glucose, the area under the time-glucose curve was used to measure the degree of change during the OGTT in each group (Figure 1D). Compared with the model group, the AUC of OGTT of SSE-L, SSE-H, and MET were significantly decreased (*p* < 0.05), and the AUC of OGTT of SSE-L was significantly lower than that of the MET group (*p* < 0.05). As shown in Figure 1E, the GSP levels were significantly lower in the normal, SSE-L, SSE-H, and MET groups compared to the model group (*p* < 0.05). In addition, the GSP levels of the SSE-L and SSE-H groups were significantly lower than in the MET group (*p* < 0.05). In addition, it was significantly lower in the SSE-L than in the SSE-H group (*p* < 0.05).

### 3.3 The effects of SSE on the islet function index and serum biochemical indices of T2DM mice

**Figure 2:**
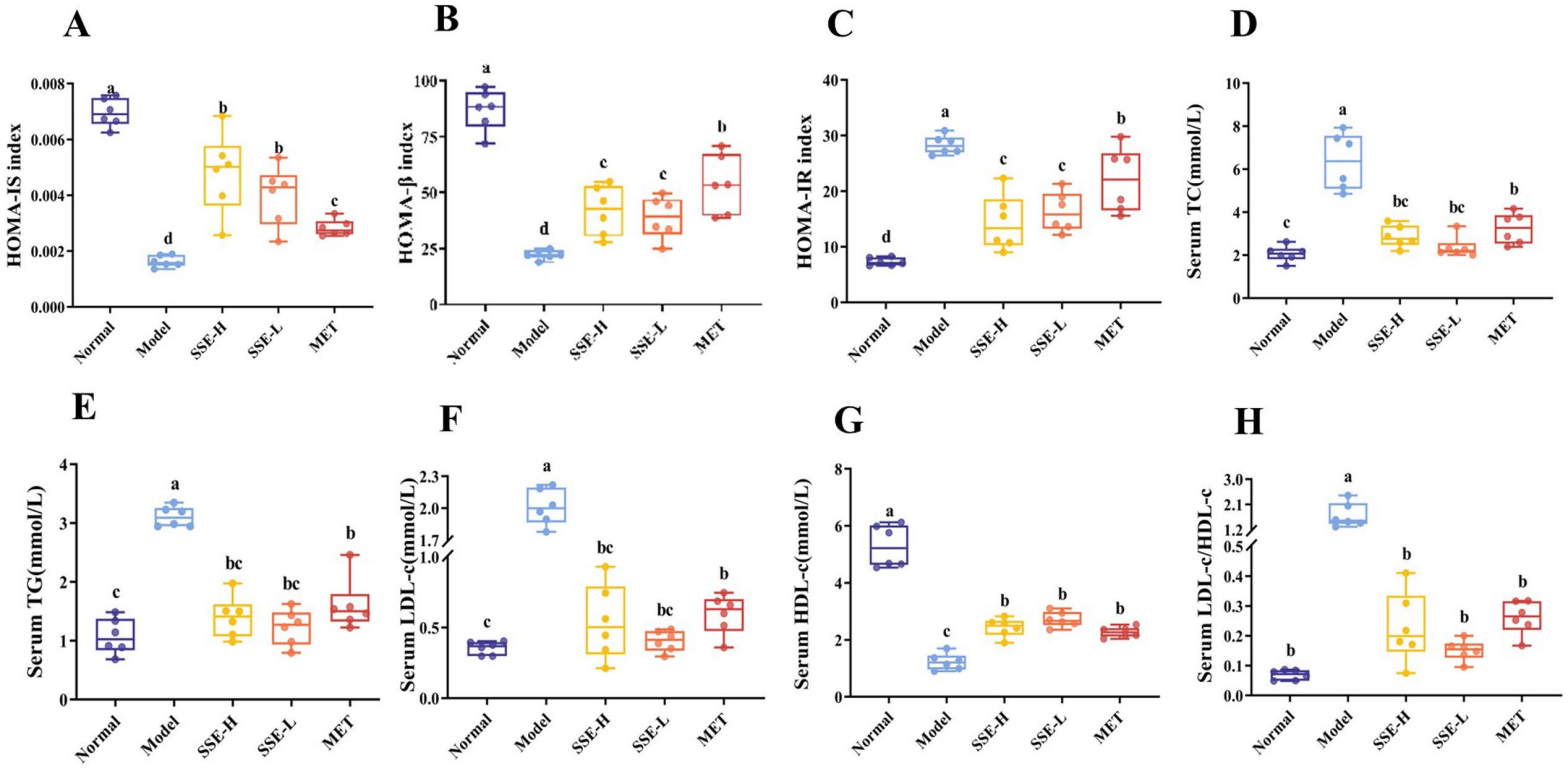
The effects of SSE on insulin correlation indices and serum biochemical indicators in T2DM model mice. **(A)** HOMA-IS, **(B)** HOMA-β, **(C)** HOMA-IR, **(D)** TC, **(E)** TG, **(F)** LDL-c, **(G)** HDL-c, **(H)** LDL-c/HDL-c. (HOMA-IS, homeostasis model assessment-insulin sensitivity index; HOMA-β, homeostasis model assessment-β index; HOMA-IR, homeostasis model assessment-insulin resistance index. Different superscript letters indicate statistically significant differences between the groups, *p* < 0.05).

HOMA is a commonly used method for assessing islet function, including islet β cell function, insulin resistance and insulin sensitivity. As shown in Figure 2A, compared with the model group, HOMA-IS index of all other groups was significantly in-creased (*p* < 0.05). Among them, the HOMA-IS index of the SSE-L and SSE-H groups was significantly higher than that of the MET group (*p* < 0.05). As shown in Figure 2B, the HOMA-β index of the normal, SSE-L, SSE-H and MET groups was significantly higher than that of the model group. In addition, the HOMA-β index of the SSE-L and SSE-H groups as significantly lower than that of the MET group (*p* < 0.05). The HOMA-IR index of the model group was significantly higher than in the other groups (*p* < 0.05, Figure 2C). Compared with the MET group, the HOMA-IR index of the SSE-L and SSE-H groups was significantly lower (*p* < 0.05). T2DM can not only cause hyperglycemia, but also cause a disorder of other serum biochemical indexes in mice. As shown in Figure 2D, the serum TC levels of the SSE-L, SSE-H and MET groups were significantly higher than in the normal group (*p* < 0.05), but were significantly lower than in the model group (*p* < 0.05). The serum TG levels of the SSE-L, SSE-H and MET groups were significantly higher than in the normal group, but were significantly lower than in the model group (*p* < 0.05, Figure 2E). As shown in Figure 2F, the serum LDL-c level of the model group was significantly higher than in the normal, SSE-L, SSE-H and MET groups (*p* < 0.05). Conversely, the serum HDL-c level of the model group was significantly lower than in the other groups (*p* < 0.05, Figure 2G). The LDL-c/HDL-c ratio is considered a predictor of metabolic disorder. As shown in Figure 2H, compared with the model group, the serum LDL-c/HDL-c ratio of the normal, SSE-L, SSE-H and MET groups was significantly decreased (*p* < 0.05).

### 3.4 The effects of SSE on liver biochemical indices, liver histopathology and mRNA levels in T2DM model mice

**Figure 3:**
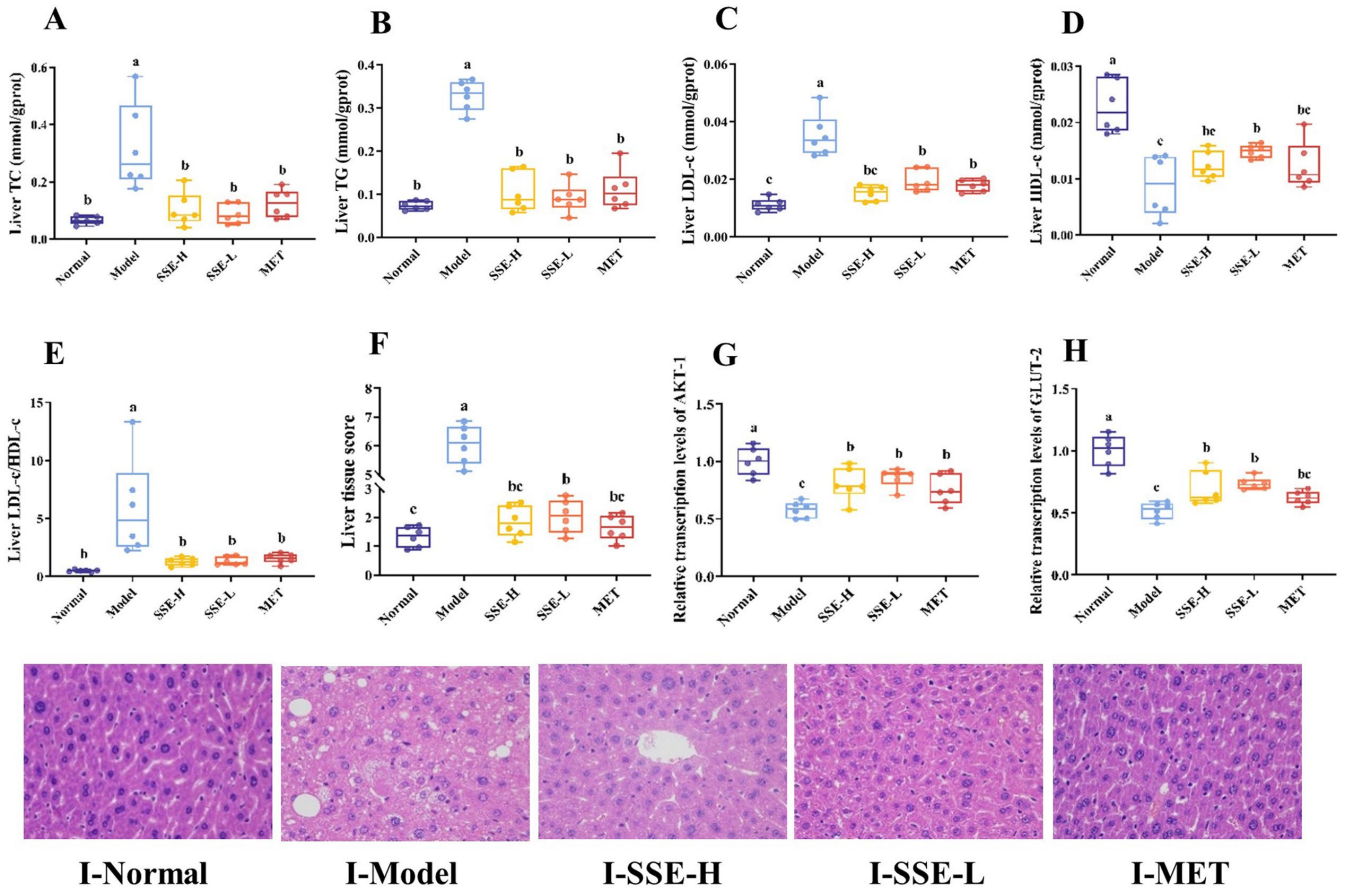
The effects of SSE on liver biochemical indexes, histopathological score, and the expression of relevant genes. **(A)** TC, **(B)** TG, **(C)** LDL-c, **(D)** HDL-c, **(E)** LDL-c/HDL-c, **(F)** liver tissue score, **(G)**AKT-1, **(H)** GLUT-2, **(I)** the stained liver tissue (400× magnification). (AKT-1, Akt serine/threonine kinase; GLUT-2, glucose transporter-2. Different superscript letters indicate statistically significant differences between the groups, *p* < 0.05).

Lipid metabolism disorders often induce the accumulation of excess lipids in the liver, which causes oxidative stress, the release of proinflammatory and proapoptotic factors, as well as liver damage to different degrees, and ultimately seriously endangers health. As shown in Figures 3A–C, the hepatic levels of TC, TG and LDL-c were significantly decreased in all treatment groups compared with the model group (*p* < 0.05). The liver LDL-c levels in the SSE-L, SSE-H and MET groups were significantly higher than in the normal group (*p* < 0.05). Conversely, the liver HDL-c levels of the SSE-L, SSE-H and MET groups were significantly lower than in the normal group (*p* < 0.05), while being significantly higher than in the model group (*p* < 0.05, Figure 3D). The liver LDL-c/HDL-c ratio was significantly decreased in all treatment groups compared to the model group (*p* < 0.05. Figure 3E). In addition, histopathological sections were examined to more clearly and intuitively demonstrate the extent of liver damage in T2DM mice (Figure 3I). In the normal group, the hepatic cords were intact and radially arranged, liver nuclei were obvious, cell structure was complete, and no histopathological changes were observed. In the model group, the arrangement of hepatic cords was disordered, presenting diffuse hepatic steatopathy, including a large number of lipid droplets inside hepatocytes, uneven size of nuclei, partial damage of liver cell membranes, and inflammatory cell infiltration. In contrast to the model group, the hepatic cords of mice in the SSE-H group were clear and neatly arranged, but there were still some lipid droplets inside hepatocytes. The liver tissue morphology of mice in the SSE-L group was significantly improved, with reduced lipid droplets and significant recovery of liver cell morphology. In addition, the arrangement of hepatic cords was disordered in the MET group, and some lipid droplets were still present. Here, we mainly investigated the following five categories of histopathological changes: degree of hepatocyte steatosis, inflammatory lesions in hepatic lobules, hepatocyte injury and liver fibrosis, hepatocyte lipid droplets and miscellaneous features. Each category was scored on a scale of 0–3 in terms of severity, with more severe changes assigned a higher score. Finally, the scores were added together to obtain the organizational score (Figure 3F). The liver tissue scores of all treatment groups were significantly decreased compared with the model group (*p* < 0.05). In addition, the liver tissue scores of the SSE-L and SSE-H groups were significantly higher than in the normal group (*p* < 0.05). In addition to histological observation, this study also investigated the effect of SSE on the relative transcription levels of Akt serine/threonine kinase (AKT-1) and GLUT-2 in the liver. The results showed that the relative transcription levels of AKT-1 and GLUT-2 in the SSE-H, SSE-L and MET groups were significantly higher than in the model group (*p* < 0.05), while being significantly lower than in the normal group (*p* < 0.05, Figures 3G,H).

### 3.5 Analysis of intestinal microbiota

**Figure 4:**
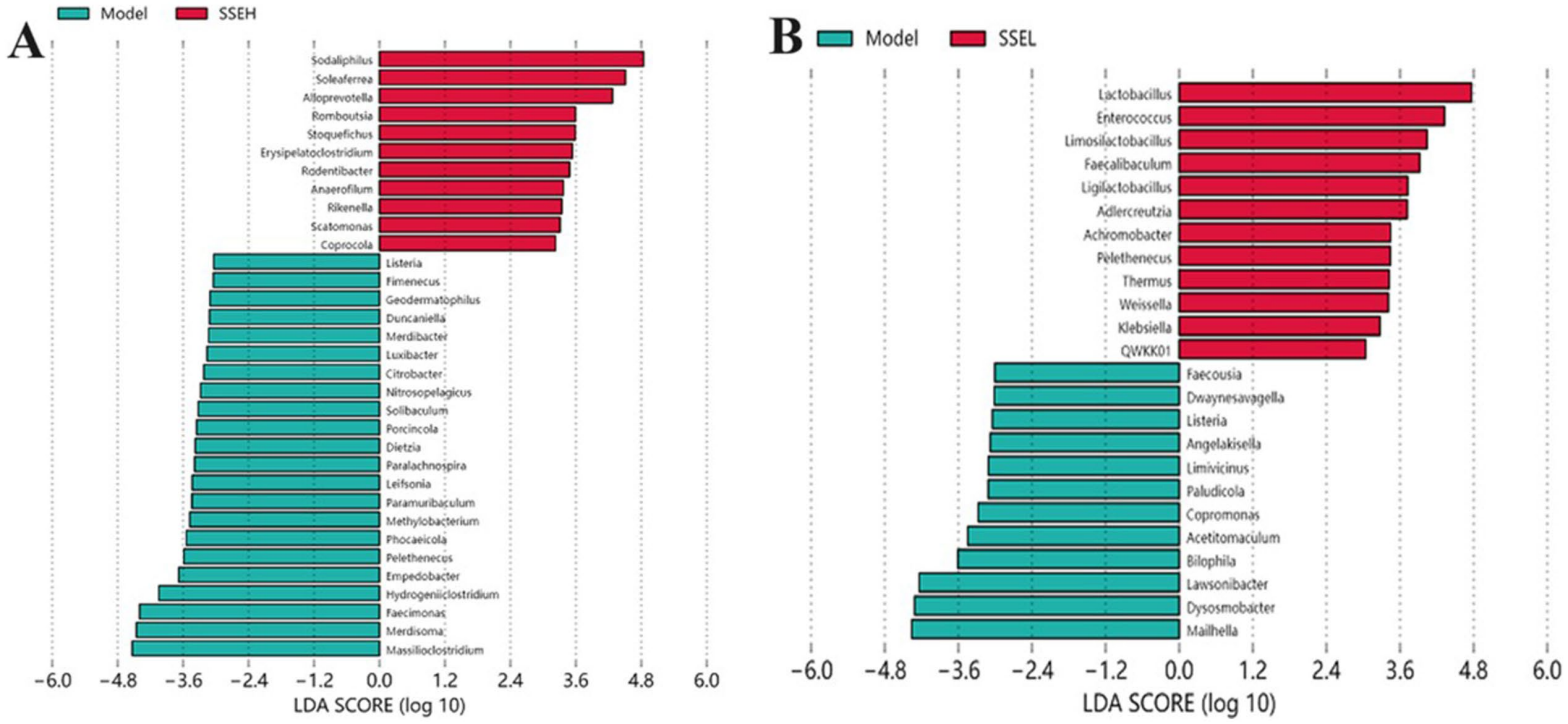
The effects of SSE on the cecal microbial community. **(A)** Model (blue) vs. SSE-H (red). **(B)** Model (blue) vs. SSE-L (red). (Different superscript letters indicate statistically significant differences between the groups, *p* < 0.05).

In this study, the bacterial genera with significant differences in the model group vs. SSE-H group and model group vs. SSE-L group were analyzed. It can be seen from Figure 4A that there were bacterial genera with significant differences between the model and SSE-H groups. Compared with the model group, the relative abundance of *Sodaliphilus*, *Soleaferrea*, *Alloprevotella*, *Romboutsia*, *Stoquefichus*, *Erysipelatoclostridium*, *Rodentibacter*, *Anaerofilum*, *Rikenella*, *Scatomonas*, and *Coprocola* was significantly increased in the in SSE-H group (*p* < 0.05). Conversely, the relative abundance of *Listeria*, *Fimenecus*, *Geodematophilus*, *Duncaniella*, *Merdibacter*, *Luxibacter*, *Citrobacter*, *Nitrosopelagicus*, *Solibaculum*, *Porcincola*, *Dietzia*, *Paralachnospira*, *Leifsonia*, *Paramuribaculum*, *Methylobacterium*, *Phocaeicola*, *Pelethenecus*, *Empedobacter*, *Hydrogeniiclostridium*, *Faecimonas*, *Merdisoma* and *Massilioclostridium* was significantly decreased (*p* < 0.05). In the SSE-L group (Figure 4B), the relative abundance of *Lactobacillus*, *Enterococcus*, *Limosilactobacillus*, *Faecalibaculum*, *Ligilactobacillus*, *Adlercreutzia*, *Achromobacter*, *Pelethenecus*, *Thermus*, *Weissella*, and *Klebsiella* was significantly increased compared to the model group (*p* < 0.05). By contrast, *Faecousia*, *Dwaynesavagella*, *Listeria*, *Angelakisella*, *Limivicinus*, *Paludicola*, *Copromonas*, *Acetitomaculum*, *Bilophila*, *Lawsonibacter*, *Dysosmobacter* and *Mailhella* exhibited the opposite trend.

### 3.6 Analysis of SCFAs and their correlation with bacterial flora and biochemical indices

**Figure 5:**
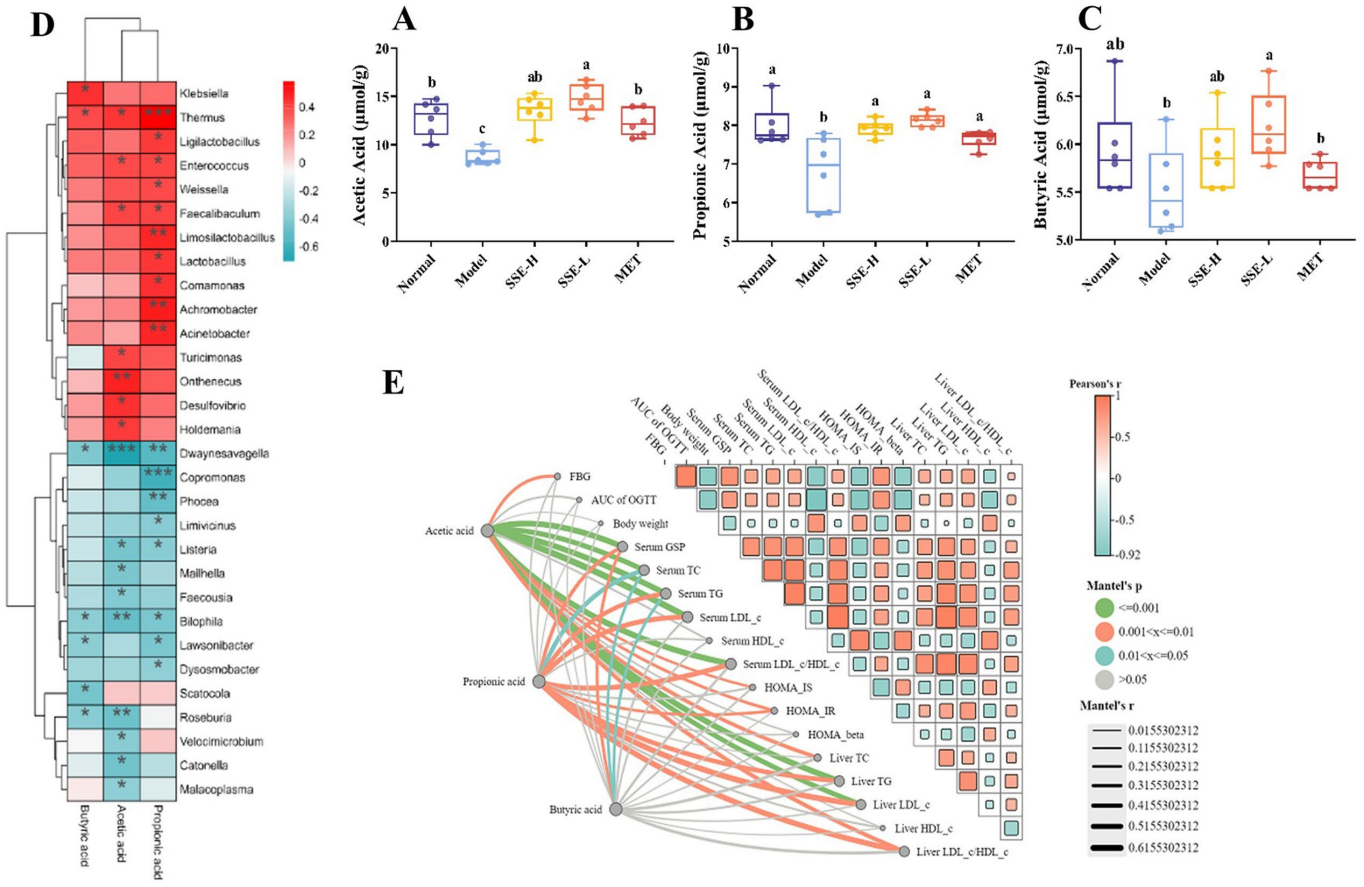
The effects of SSE intervention on the levels of SCFAs in the cecum contents of T2DM mice and the correlation between bacterial genera and hypoglycemic parameters. **(A)** Acetic acid, **(B)** propionic acid, **(C)** butyric acid, **(D)** the correlation heat map between bacterial genera and SCFAs, Red indicates positive correlation, and blue indicates negative correlation, **(E)** the correlation between hypoglycemic parameters and SCFAs. Orange indicates positive correlation, and blue indicates negative correlation. The thicker the line, the larger the circle, the more obvious the difference. [SCFAs, short-chain fatty acids. Different superscript letters indicate statistically significant differences between the groups (*p* < 0.05)].

**Figure 6:**
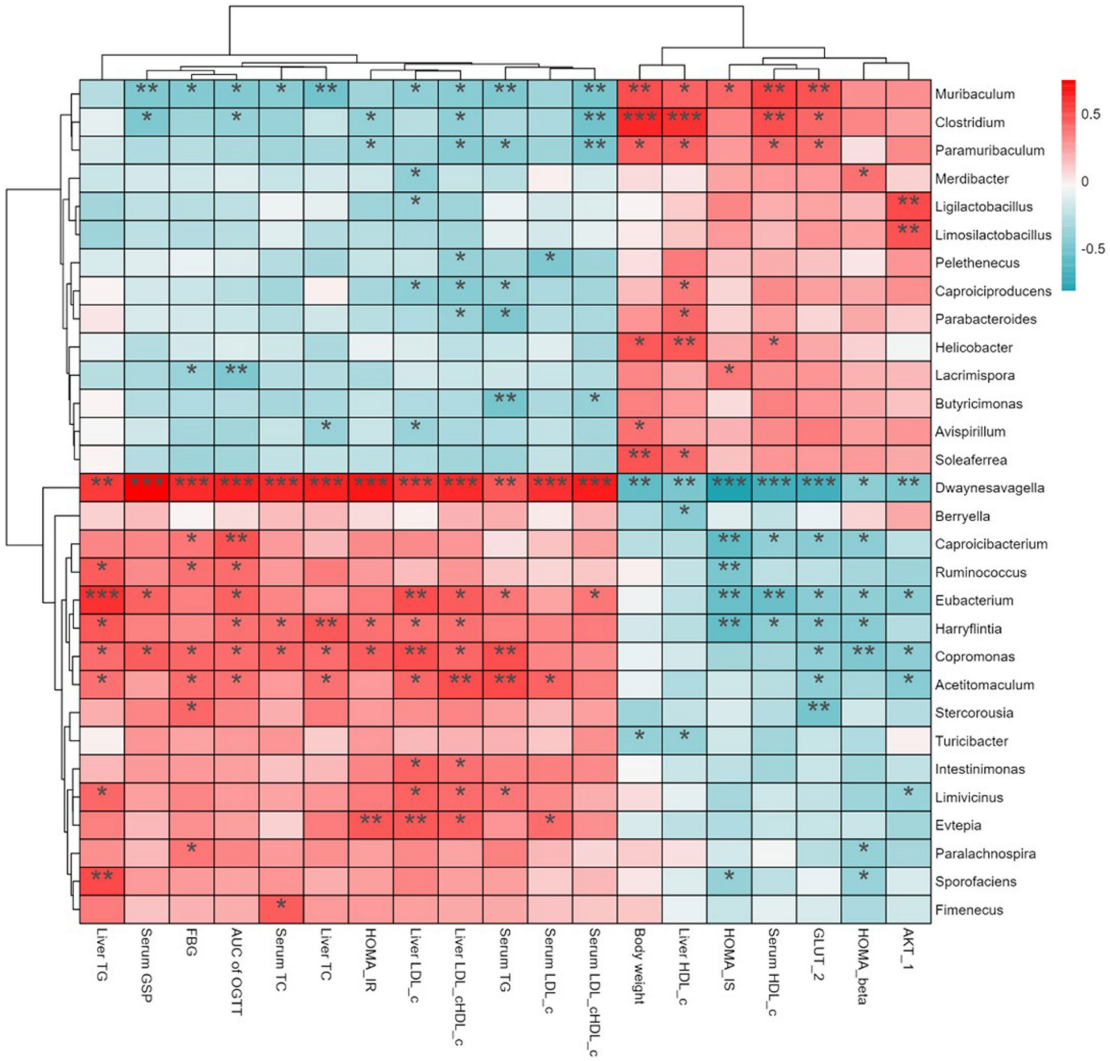
Heatmap showing the correlation between bacterial genera hypoglycemic parameters (red indicates positive correlation, and blue indicates negative correlation. Asterisks (*) indicate the strength of the correlation).

**Figure 7:**
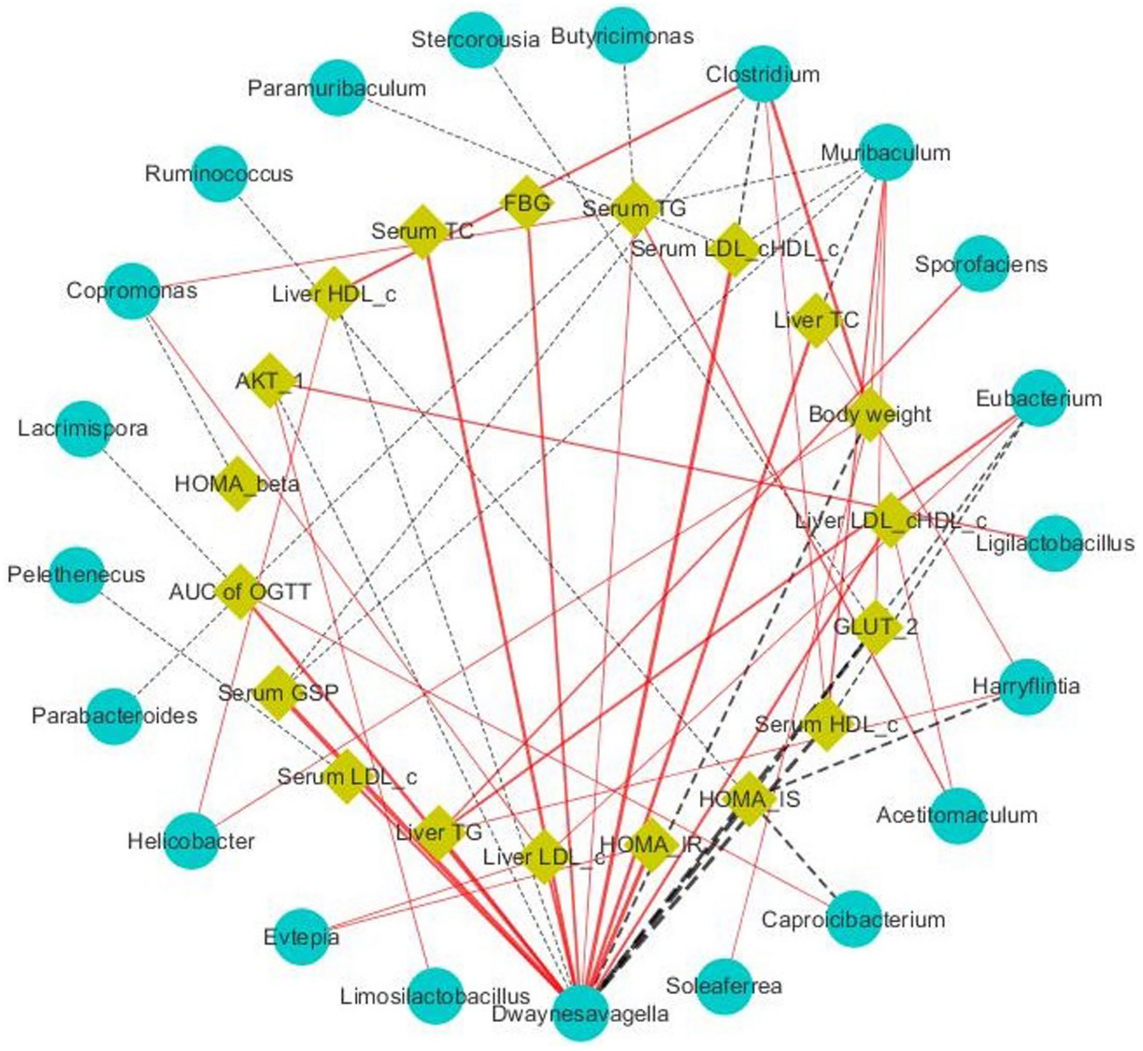
Visualization of the correlation network according to the partial correlation between the intestinal microbiota and glucose metabolic parameters. The yellow and blue nodes represent the glucose metabolism disorder parameters and intestinal microbiota at the genus level, respectively. The solid red lines and dotted black lines represent positive and negative correlations, respectively, while the line width indicates the strength of correlation. Only the significant edges (Spearman correlation test |*r*| > 0.4, FDR adjusted *p* < 0.01) were drawn in the network.

Short-chain fatty acids (SCFAs), which mainly include acetic, propionic, and butyric acid, are a class of metabolites produced by the microbial fermentation of carbohydrates in the intestines. Figures 5A–C show the changes of SCFA content in the intestines of T2DM mice after SSE treatment. Compared with the model group, the contents of acetic, propionic and butyric acid in the SSE-L and SSE-H groups were significantly increased (*p* < 0.05). It is worth noting that only the acetic acid content of the SSE-L group was significantly higher than that of the MET group (*p* < 0.05, Figure 5A). In order to further understand the relationship of intestinal microbiota with acetic, propionic and butyric acid, we mapped the correlation and analyzed a heatmap at the genus level. As can be seen in Figure 5D, *Thermus* was significantly positively correlated with acetic, propionic and butyric acid (*p* < 0.05), while *Dwaynesavagella* and *Bilophila* were significantly negatively correlated (*p* < 0.05). In addition, *Enterococcus* and *Faecalibaculum* were significantly positively correlated with acetic and propionic acid (*p* < 0.05), while Listeria was significantly negatively correlated (*p* < 0.05). Mantel test analysis was used to determine the correlation between hypoglycemic parameters and SCFAs (Figure 5E). Hypoglycemic parameters such as GSP, serum TC, and serum TG were associated with acetic acid, propionic acid, and butyric acid (*p* < 0.001). This study also analyzed the correlation between representative bacterial genera and related indexes of hypoglycemia after 4 weeks of intervention with different doses of SSE (Figure 6). It is worth noting that *Dwaynesavagella* was significantly correlated with all the tested hypoglycemic indexes. It was significantly positively correlated with liver TG, GSP, FBG, AUC of OGTT, serum TC, liver TC, HOMA-IR, liver LDL-c, liver LDL-c/HDL-c, serum TG, serum LDL-c and serum LDL-c/HDL-c. Conversely, it was significantly negatively correlated with body weight, liver HDL-c, HOMA-IS, serum HDL-c, GLUT-2, HOMA-β and AKT-1 (*p* < 0.05). FBG is the most intuitive indicator of blood glucose levels, and it showed a significant positive correlation with *Paralachnospira*, *Stercorousia*, *Acetitomaculum*, *Copromonas*, *Ruminococcus*, *Paralachnospira*, as well as *Caproicibacterium* (*p* < 0.05). Conversely, it exhibited a significant negative correlation with *Lacrimispora* and *Muribaculum* (*p* < 0.05). As shown in Figure 7, parameters with a correlation coefficient |*r*| ≥ 0.45 were screened. *Dwaynesavagella* demonstrated a strong negative correlation with HOMA-IS, serum HDL-c, GLUT-2, body weight, liver HDL-c, and AKT-1 (*r* = −0.814, −0.686, −0.676, −0.567, −0.471, and −0.468, respectively), as well as a positive correlation with serum TG, liver TG, liver LDL-c, serum LDL-c, liver LDL-c/HDL-c, FBG, serum TC, AUC of OGTT, liver TC, serum LDL-c/HDL-c, HOMA-IR, and GSP (*r* = 0.465, 0.559, 0.571, 0.587, 0.599, 0.6, 0.605, 0.629, 0.63, 0.651, 0.668, and 0.753, respectively). In addition, *Clostridium* demonstrated a strong negative correlation with serum LDL-c/HDL-c and GSP (*r* = −0.503 and −0.456, respectively) as well as a strong positive correlation with serum HDL-c, liver HDL-c, and body weight (*r* = 0.492, 0.583, and 0.624, respectively). Additionally, *Caproicibacterium* demonstrated a strong negative correlation with HOMA-IS (*r* = −0.568) and a strong positive correlation with the AUC of OGTT (*r* = 0.483).

## 4 Discussion

Globally, about one in 11 adults has diabetes, with 90% of the cases being diagnosed as T2DM. With Asia at the epicenter of the epidemic, T2DM is also a major health problem for China. There are several strategies to treat T2DM, such as antidiabetic medications, dietary interventions and lifestyle changes. Although the commonly used oral antidiabetic drugs are effective, side effects are inevitable. Chemical drugs such as metformin and rosiglitazone can cause great damage to the health of patients (29). To date, the treatment of T2DM remains extremely challenging, and a growing number of studies suggest that intervention with natural compounds (probiotics, prebiotics, polyphenols, polysaccharides and flavonoids, etc.) is a potential strategy to effectively prevent and manage T2DM (30). *Suaeda salsa* is very rich in active compounds, including minerals, polysaccharides, flavonoids and phenols. The in-depth study of these substances revealed prominent pharmacological effects, such as regulating blood sugar, as well as anti-inflammatory, anti-aging and antimicrobial activities (31). Therefore, this study investigated the effects of ethanolic extract of *Suaeda salsa* on hyperglycemia and intestinal flora in T2DM model mice.

In this study, different doses of SSE were continuously administered toT2DM mice for 4 weeks, and the results showed that compared with the normal group, the body weight decreased significantly in all treatment groups (*p* < 0.05). This may be because after successful modeling, T2DM will lead to impaired energy metabolism and decreased utilization of carbohydrates, finally resulting in weight loss. After 4 weeks of SSE treatment, the body weight of mice in the SSE-L group increased significantly compared with the model group (*p* < 0.05), indicating that SSE can promote the absorption and utilization of carbohydrates in T2DM mice, thereby alleviating abnormal weight loss. By contrast, the weight of mice in the MET group showed no obvious trend of recovery. This may be due to the fact that MET inhibits the intestinal absorption and utilization of glucose, leading to weight loss. The most intuitive way to diagnose T2DM is to examine the FBG levels. After the successful establishment of the T2DM mouse model, the FBG value increased rapidly, while after 2 and 4 weeks of intervention, it significantly decreased in the SSE-H, SSE-L and MET groups, which preliminarily indicated that SSE and MET have a certain lowering effect on the blood glucose levels of the mice. Notably, the effect of SSE-L was better than that of MET. However, FBG can only reflect the instantaneous blood glucose value. In order to ensure the accuracy of diagnosis, more effective detection methods are needed. OGTT is used to detect the body’s ability to regulate blood sugar and reflects the function of is-let β cells (32). The AUC of OGTT can be used to modify the OGTT test by comprehensively calculating the blood glucose values at 4 time points during the OGTT process. In this study, the blood glucose levels of mice in all groups rose sharply after glucose administration, reached their peak within 0.5 h, and subsequently showed a downward trend. However, the blood glucose level of the model group was significantly higher than in all other groups, among which the blood glucose levels of the SSE-H, SSE-L and MET groups were significantly lower at all time points (*p* < 0.05). This indicates that T2DM leads to impaired glucose tolerance in mice, which can be ameliorated to a certain extent by SSE treatment. The AUC of OGTT analysis showed that the glucose tolerance of the MET, SSE-H and SSE-L groups was significantly higher than in the model group (*p* < 0.05), whereby SSE-L showed a more significant improvement. Glycosylated serum protein (GSP) levels are not affected by transient blood sugar fluctuations, and reliably reflects a patient’s average blood sugar level over the preceding 1–3 weeks (33). In this study, the GSP levels of the SSE-L and SSE-H groups were significantly lower than in the MET group (*p* < 0.05), and the GSP level of the SSE-L group was significantly lower than that of the SSE-H group (*p* < 0.05). These results indicate that the effect of SSE is dose-dependent.

The early stage of T2DM is usually characterized by insulin resistance, in which the body’s sensitivity to insulin decreases, resulting in increased blood glucose levels after meals. In order to balance the high concentration of blood sugar, the islet β cells will continue to produce insulin compensatively, further increasing their workload, ultimately impacting pancreatic function (34). As a comprehensive evaluation method for islet function, HOMA can reflect the function of islet β cells and insulin sensitivity. In this study, the levels of HOMA-IS and HOMA-β were significantly higher in the SSE-L, SSE-H and MET groups than in the model group (*p* < 0.05). By contrast, HOMA-IR was significantly lower in the SSE-L, SSE-H and MET groups than in the model group (*p* < 0.05). Hence, HOMA-IS and HOMA-β increase with insulin sensitivity, but HOMA-IR exhibited the opposite trend. However, the treatment effect of SSE-H and SSE-L was significantly better than that of MET. In summary, insulin sensitivity improved after 4 weeks of treatment using either conventional MET or different concentrations of SSE. Chronic hyperglycemia is a serious metabolic disorder that interferes with carbohydrate and lipid metabolism. Accordingly, the loss of blood glucose regulation in T2DM patients is often accompanied by abnormal blood lipid levels (35). In this study, the levels of serum TC, TG, LDL-c and LDL-c/HDL-c were significantly lower in the SSE-H, SSE-L and MET groups than in the model group. However, HDL-c exhibited the opposite trend. The serum levels of TC, TG and LDL-c were significantly lower in SSE-L group than in the MET group (*p* < 0.05), which was consistent with Abeyrathna and Su (36). These results indicate that the T2DM model mice exhibited obvious lipid metabolism disorder and dyslipidemia. However, after 4 weeks of treatment with SSE, the blood lipid levels improved in all groups. In conclusion, different concentrations of SSE can improve the lipid metabolism abnormalities associated with T2DM, whereby the effect was dose-dependent.

Nonalcoholic fatty liver disease (NAFLD), which is commonly associated with type 2 diabetes, eventually leads to liver injury due to metabolic stress. In this study, the hepatic levels of TC, TG, and LDL-c as well as the LDL-c/HDL-c ratio, were significantly decreased in the SSE-L and SSE-H groups compared with the model group (*p* < 0.05). By contrast, HDL-c exhibited the opposite trend, which was largely consistent with the serum related indicators, indicating that SSE had the ability to ameliorate the dysfunction of liver lipid metabolism. Hematoxylin-eosin (H&E) staining is the most widely used technique in histology, embryology and pathology studies. In this study, the liver histology of the model group was disordered, with a large number of lipid droplets inside hepatocytes. By contrast, the hepatic cord arrangement of the SSE-H and MET groups was relatively orderly, and lipid droplets were significantly reduced. In the SSE-L group, liver tissue morphology was significantly improved, lipid droplets were significantly reduced, and liver cell morphology was largely restored, indicating that both SSE and MET can effectively inhibit hepatic lipid accumulation. Moreover, SSE-L may be more effective than conventional MET treatment in preventing the progression of NAFLD in T2DM mice. AKT-1 is a critical signaling pathway involved in vascular cell survival, proliferation, migration, as well as the regulation of lipid and glucose metabolism (37). Glucose transporter-2 (GLUT-2) has captured the attention of many scholars due to its tissue specificity and complexity of gene expression regulation. Under the influence of metabolic diseases such as diabetes, GLUT-2 undergoes compensatory functional regulation, complicating its gene regulation (38, 39). In this study, the expression levels of AKT-1 and GLUT-2 were significantly higher the in SSE-H, SSE-L and MET groups than in the model group (*p* < 0.05), indicating that these two genes play an important role in controlling the lipid metabolism of T2DM mice.

T2DM can lead to severe microbiota imbalance, and dietary intervention is an effective strategy to regulate the gut microbiota (40). There is evidence of significant differences in the composition of intestinal microbiota between healthy and diabetic individuals, and these differences may be related to the pathogenesis of metabolic diseases (41). In this study, there were significant differences in the relative abundance of numerous bacterial genera in both the SSE-H and SSE-L groups (*p* < 0.05). Among them, *Candidatus Soleaferrea* is linked with intestinal inflammation and generalized persistent low-level inflammation. The activation of the immune system, leading to an inflammatory response, is linked with the development of T2DM, thereby establishing that increased abundance of the genus *Soleaferrea* is a risk factor for T2DM. *Alloprevotella* is capable of producing SCFAs, and its increased abundance is associated with reduced inflammation, ameliorating nonalcoholic fatty liver disease and treating T2DM (42, 43). The species *Phocaeicola vulgatus* (formerly *Bacteroides vulgatus*) was found to exhibit decreased abundance in diabetics (44). *Lactobacillus*, which is highly abundant in the human gut, is a potential probiotic that regulates liver damage and insulin resistance symptoms in diabetic mice (45). In addition, we found that *Faecalibaculum* exhibited decreased abundance in the diabetes model, but significantly rebounded following treatment with SSE-L, which was consistent with the therapeutic effect found by Xu et al. (46) in TFCP. *Bilophila wadsworthia* is a major representative of sulfidogenic bacteria in the human gut. It was originally recovered in different clinical specimens of intraabdominal infections and was recently reported to be potentially linked with different chronic metabolic disorders. Consistent with the results of this study, *Bilophila* was found to be significantly positively correlated with the development of diabetes and diabetic kidney disease (47, 48). Therefore, we can speculate that the abundant strains in the SSE group may alleviate the symptoms of diabetes, while the significantly decreased strains may conversely promote the development of diabetes.

SCFAs, mainly including acetic, propionic and butyric acid, are produced in the fermentation of dietary fibers by intestinal microbiota. They can affect glucose metabolism and insulin sensitivity through various pathways, thus affecting the development of diabetes (49). The level of intestinal SCFAs was significantly decreased in the model group, while being significantly increased after SSE and MET treatment (*p* < 0.05). The acetic acid content of the SSE-L group was significantly higher than that of the MET group (*p* < 0.05). This may be due to changes in the structure and function of intestinal microflora in T2DM mice, consistent with the findings of Wang (50). Further analysis of the correlation between intestinal microorganisms and SCFAs showed that *Thermus* was significantly positively correlated with acetic, propionic and butyric acid (*p* < 0.05), while *Dwaynesavagella* and *Bilophila* were significantly negatively correlated with acetic, propionic and butyric acid (*p* < 0.05). In addition, *Enterococcus* and *Faecalibaculum* were significantly positively correlated with the acetic and propionic acid levels (*p* < 0.05), while *Listeria* exhibited a significant negative correlation (*p* < 0.05). Therefore, it can be inferred that SSE improved the abundance of health-promoting intestinal microbes, which in turn increased the SCFA levels, thereby ameliorating the symptoms of hyperglycemia in mice. Mantel test analysis revealed that GSP, serum TC, and serum TG were associated with acetic, propionic, and butyric acid levels (*p* < 0.001). Thus it can be concluded that SSE can be decomposed and utilized by intestinal microorganisms, regulate the intestinal microbial community structure, increase the abundance of acid-producing bacteria, promote the production of SCFAs, and thereby regulate GSP, serum TC, serum TG, and other related indexes of T2DM mice [51]. Notably, *Dwaynesavagella* was significantly correlated with all the indexes analyzed in this study (*p* < 0.05), and this genus exhibited a significant decrease in relative abundance (*p* < 0.05). Based on the research results of this study, we can preliminarily conclude that SSE can reduce the relative abundance of *Dwaynesavagella* and increase the content of SCFAs in the intestine, thereby reducing blood glucose levels, improving serum and liver lipid homeostasis and islet function, thereby ultimately ameliorating the symptoms of diabetic mice.

## 5 Conclusion

In this study, a mouse model of T2DM was constructed by administering a high-sugar/high-fat diet combined with intraperitoneal injection of streptozotocin to analyze the effects of SSE on body weight, blood glucose levels, islet function, serum and liver biochemical indices, as well as the intestinal microbiota. The results showed that SSE could improve the body weight, blood glucose levels, islet index as well as the blood and liver biochemical indexes of mice, in a dose-dependent manner. SSE also increased the intestinal content of SCFAs *in vivo*, increase the abundance of beneficial bacteria, and reduce the abundance of harmful bacteria. As a consequence, it reduced the blood glucose levels of the T2DM model mice. The correlation results showed that *Dwaynesavagella* may be a bacterial marker for type 2 diabetes. Overall, the results of this study provide a theoretical basis for further understanding the effect of SSE on hyperglycemia symptoms in T2DM.

## Data Availability

The original contributions presented in the study are included in the article/Supplementary material, further inquiries can be directed to the corresponding author.

## Footnotes

^1^ https://bioincloud.tech/about-us

## Supplementary material

SUPPLEMENTARY FIGURE S1 The negative UPLC-QTOF-MS/MS map of SSE.

SUPPLEMENTARY FIGURE S2 The positive UPLC-QTOF-MS/MS map of SSE.

SUPPLEMENTARY TABLE S1 The main compounds of SSE using UPLC-QTOF/MS.
