# Supplementary figures and images for: Regulation of intestinal flora by Suaeda salsa extract ameliorates hyperglycemia in a mouse model of type 2 diabetes mellitus
Source: Front Nutr. 2024 Dec 16;11:1499196. doi: 10.3389/fnut.2024.1499196 (PMC11682902; doi:10.3389/fnut.2024.1499196)

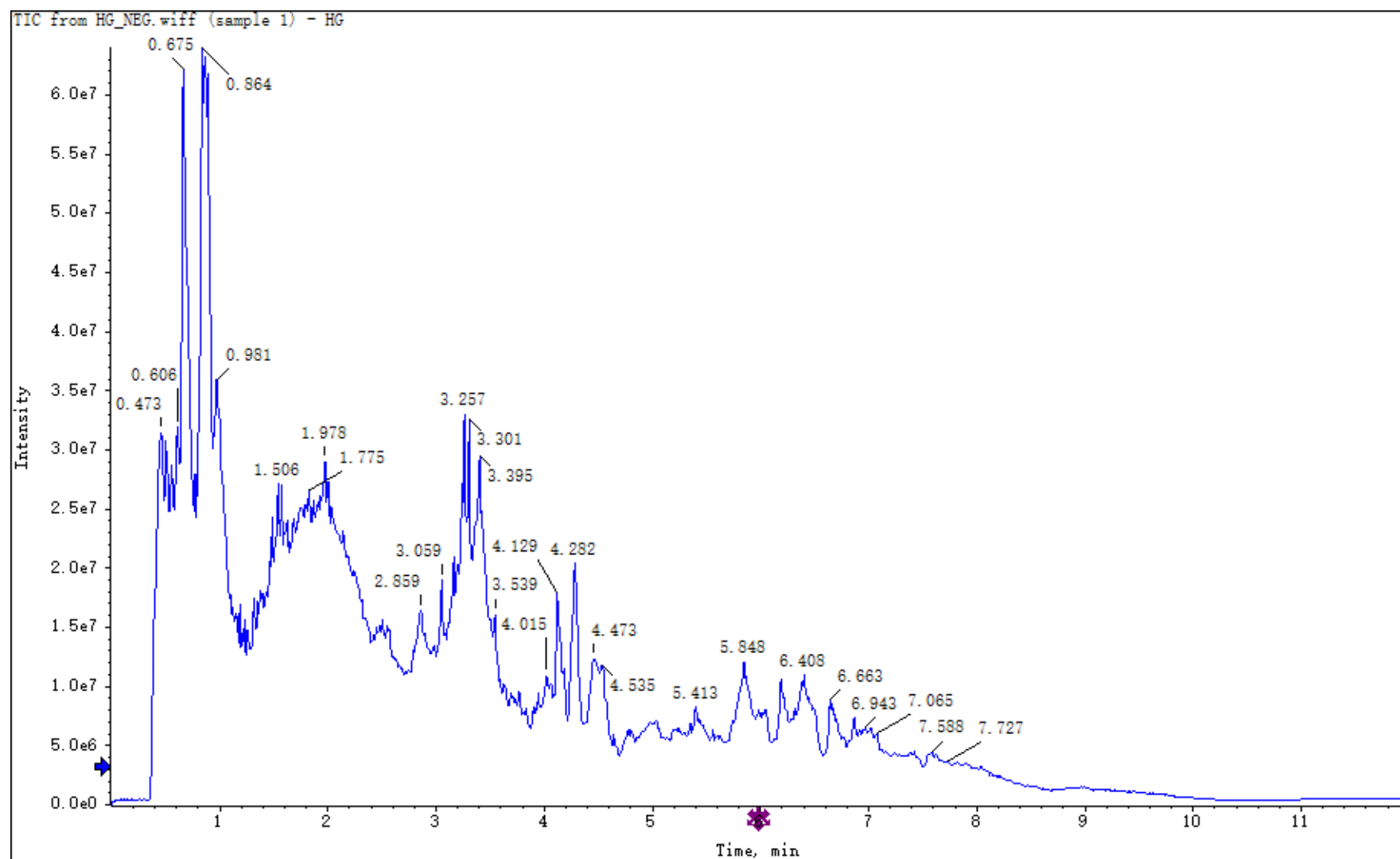

Supplement: Supplementary file 1 [file Data_Sheet_1.ZIP › Supplementary Files/Figure S1-NEG.pdf]

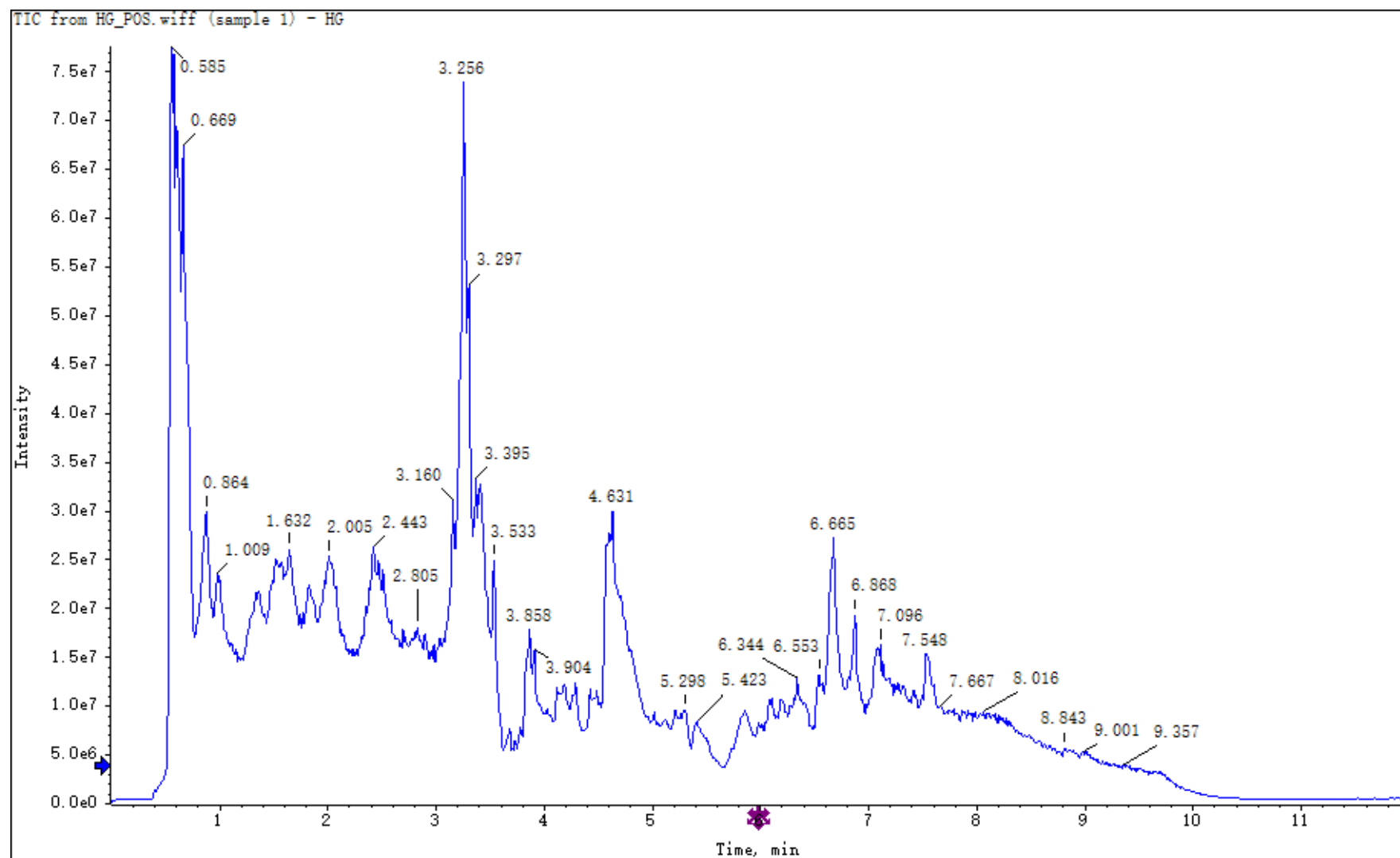

Supplement: Supplementary file 1 [file Data_Sheet_1.ZIP › Supplementary Files/Figure S2-POS.pdf]
